# Intermediate-Term Outcomes of Endoscopic or Open Vein Harvesting for Coronary Artery Bypass Grafting: The REGROUP Randomized Clinical Trial

**DOI:** 10.1001/jamanetworkopen.2021.1439

**Published:** 2021-03-15

**Authors:** Marco A. Zenati, Deepak L. Bhatt, Eileen M. Stock, Brack Hattler, Todd H. Wagner, Faisal G. Bakaeen, Kousick Biswas

**Affiliations:** 1 Division of Cardiac Surgery, Department of Surgery, Veterans Affairs Boston Healthcare System, Brigham and Women’s Hospital, Harvard Medical School, Boston, Massachusetts; 2 Brigham and Women’s Hospital, Harvard Medical School, Boston, Massachusetts; 3 Cooperative Studies Program, Perry Point/Baltimore Coordinating Center, Office of Research and Development, US Department of Veterans Affairs, Perry Point, Maryland; 4 VA Eastern Colorado Healthcare System, Denver; 5 VA Health Economics Resource Center, Department of Surgery, Stanford University, Palo Alto, California; 6 Cleveland Clinic, Cleveland, Ohio; 7 Perry Point Cooperative Studies Program Coordinating Center, Office of Research and Development, US Department of Veterans Affairs, Perry Point, Maryland; 8 Department of Epidemiology and Public Health, University of Maryland School of Medicine, Baltimore

## Abstract

This randomized clinical trial examines intermediate-term outcomes of endoscopic vs open vein harvesting for coronary artery bypass grafting as part of the Randomized Endo-Vein Graft Perspective (REGROUP) trial.

## Introduction

Endoscopic vein harvesting (EVH) for coronary artery bypass grafting (CABG) was introduced in the 1990s to reduce the rates of leg wound complications.^1^ Technical mastery of EVH requires a significant learning curve.^2^ In 2009, a study in 3000 patients raised the concern that, compared with conventional harvesting, EVH was associated with a 50% increase in mortality.^3^ With the aim of assessing the safety of EVH, we report the intermediate-term results of the Randomized Endo-Vein Graft Prospective (REGROUP) trial.^4,5^

## Methods

The randomized clinical trial was approved by the institutional review board at each participating center. Patients gave written informed consent before participation. This study is reported following the Consolidated Standards of Reporting Trials (CONSORT) reporting guideline. The trial protocol and statistical analysis plan are available in Supplement 1. This study is registered on ClinicalTrials.gov (Identifier: NCT01850082).

In the Randomized Endo-Vein Graft Perspective (REGROUP) trial, participants undergoing CABG at 16 Department of Veterans Affairs (VA) medical centers from March 2014 through April 2017 were randomized to either EVH or open vein harvest (OVH) in a 1:1 ratio. The primary outcome of the REGROUP trial was defined as the first occurrence of a major adverse cardiac event (MACE) composite comprised of all-cause mortality, nonfatal myocardial infarction (MI), or repeat revascularization, over the active follow-up phase; we previously reported no difference in MACE between the 2 groups, with a median follow-up of 2.7 years.^5^ The prespecified secondary outcome was the first occurrence of MACE over the combined active and passive follow-up phases (eAppendix in Supplement 2). Only expert EVH harvesters (ie, individuals who had performed >100 EVHs with <5% conversions to OVH) were allowed to perform the procedures. Inclusion criteria for patients were age 18 years or older, elective or urgent CABG, and plan to undergo at least 1 CABG using a saphenous-vein graft. Assessments occurred at baseline, intraoperatively, postoperatively, at discharge or 30 days after surgery if still hospitalized, at 6 weeks, and every 3 months thereafter until the end of the active follow-up phase; intermediate-term MACE outcomes were collected passively for an additional 2 years by merging VA and non-VA records as well as Medicare parts A and B records. Sample size calculations estimated a need for a total of 1150 patients. Kaplan-Meier nonparametric survival estimates depicted the unadjusted impact of vein harvesting technique on MACE. Multivariable survival analyses were performed. *P* values were 2-sided, and statistical significance was set at *P* = .05. Analyses were conducted using SAS statistical software version 9.4 (SAS Institute). Data were analyzed in August 2020.

## Results

**Figure 1.**
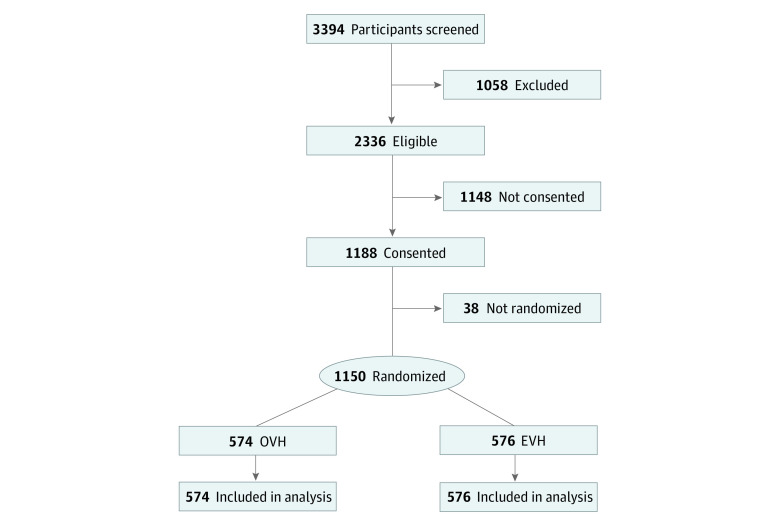
Participant Enrollment and Randomization Flowchart One ineligible participant was mistakenly considered eligible and was consented and randomized.

**Figure 2.**
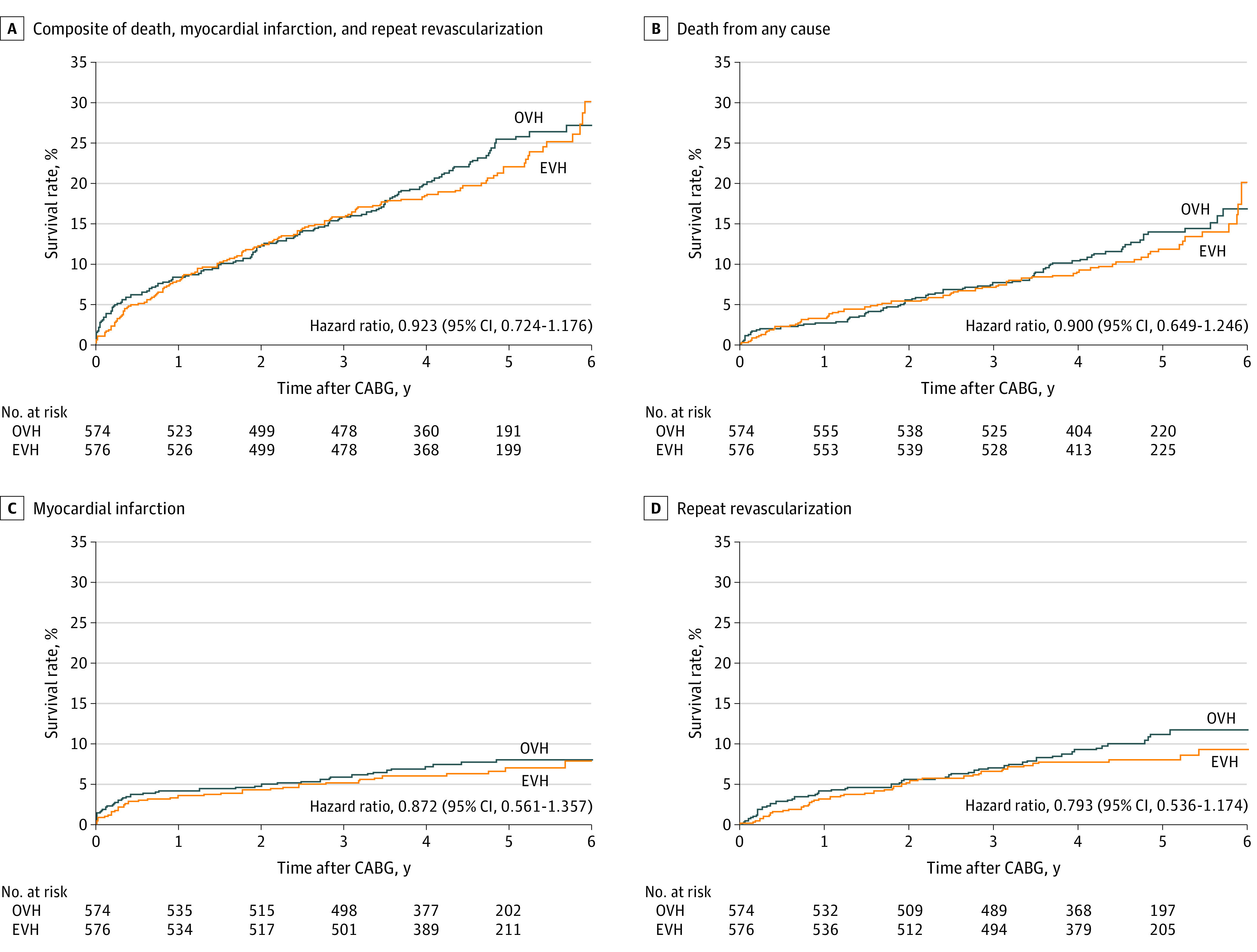
Kaplan-Meier Estimates of Rates of Survival and Major Adverse Cardiac Events After Coronary Artery Bypass Graft (CABG) EVH indicates endoscopic vein harvesting; OVH, open vein harvesting.

A total of 1150 participants (mean [SD] age, 66.4 [6.9] years; 1143 [99.5%] men) were randomized, including 574 patients (49.9%) randomized to OVH and 576 patients (50.1%) randomized to EVH, and followed-up for MACE (Figure 1). Groups were balanced regarding age, sex, smoking status, race/ethnicity, body mass index, and comorbid conditions.^5^ The median (interquartile range) intermediate-term follow-up was 4.70 (3.84-5.45) years. During the entire follow-up, the primary outcome of MACE occurred in 126 patients (21.9%) in the EVH group, compared with 135 patients (23.5%) in the OVH group (hazard ratio [HR], 0.92; 95% CI, 0.72-1.18; *P* = .52) (Figure 2). Death from any cause occurred in 69 patients (12.0%) in the EVH group and 76 patients (13.2%) in the OVH group (HR, 0.90; 95% CI, 0.65-1.25; *P* = .52); nonfatal MI occurred in 37 patients (6.4%) in the EVH group and 42 patients (7.3%) in the OVH group (HR, 0.87; 95% CI, 0.56-1.36; *P* = .54); and repeat revascularization occurred in 45 patients (7.8%) in the EVH group and 56 patients (9.8%) in the OVH group (HR, 0.79; 95% CI, 0.54-1.17; *P* = .25). An adjusted multivariable Cox regression model found no significant difference in MACE risk.

## Discussion

This randomized clinical trial found that there was no significant difference in MACE occurrence among patients who underwent EVH compared with those who underwent OVH for CABG over a median follow-up of 4.7 years. The saphenous vein is the most common supplementary conduit for CABG, but concerns have been raised about long-term ischemic events when EVH is used. This uncertainty has translated into variable adoption rates of EVH in North America (>80% of patients) compared with Europe (<50% of patients).^6^

The intermediate-term results of REGROUP are reassuring and demonstrate no significant difference in cardiovascular events between endoscopic or open approaches; leg-wound complications were reduced with EVH.^5^ Limitations of this study include lack of imaging evaluation of graft patency and use of only expert harvesters. These results provide strong reassurance that EVH is safe up to 4.7 years after the procedure; a 10-year follow-up is planned.

## Notes

Supplement 1. **Trial Protocol and Statistical Analysis Plan**

Supplement 2. **eAppendix.** Supplementary Methods

Supplement 3. **Data Sharing Statement**
