# Supplementary material for: Intermediate-Term Outcomes of Endoscopic or Open Vein Harvesting for Coronary Artery Bypass Grafting: The REGROUP Randomized Clinical Trial
Source: JAMA Netw Open. 2021 Mar 15;4(3):e211439. doi: 10.1001/jamanetworkopen.2021.1439 (PMC7961312; doi:10.1001/jamanetworkopen.2021.1439)
Supplement: Supplement 2. — eAppendix. Supplementary Methods [file jamanetwopen-e211439-s002.pdf]

Supplemental Online Content

Zenati MA, Bhatt DL, Stock EM, et al. Intermediate-term outcomes of endoscopic or open vein harvesting for coronary artery bypass grafting: the REGROUP randomized clinical trial. *JAMA Netw Open*. 2021;4(3):e211439. doi:10.1001/jamanetworkopen.2021.1439

eAppendix. Supplementary Methods

This supplemental material has been provided by the authors to give readers additional information about their work.

© 2021 Zenati MA et al. *JAMA Network Open.*

eAppendix. Supplementary Methods

Active Follow-Up Phase

Every three months during the active follow-up period post-surgery, the site study coordinator will contact the study subjects by telephone to determine whether they have experienced any problems and/or procedures related to their cardiac health (i.e., acute myocardial infarctions, revascularization procedures, or a clinically indicated cardiac catheterization). These interim calls will collect major adverse cardiac events (MACE) data, maintain rapport with subjects and let them know that the study team at the sites is interested in their progress. At one year the subject will complete Quality of Life surveys by telephone or mail.

Passive Follow-up Phase

Long-term secondary MACE outcomes will be collected by passive follow-up using VA databases for an additional two years after the completion of the active follow-up phase. At the end of the active follow-up phase (approximately 4.5 years from study start-up) each continuing subject's status will be recorded in a termination form to indicate official termination of the active follow-up phase of the study. All subjects terminated from the active follow-up phase of the study will be continually followed by the study's national nurse coordinator where existing VA administrative databases will be mined for MACE for another two years (passive follow-up phase). In addition, VA Health Economics Resource Center (HERC) personnel will merge databases including VA-purchased care files, Medicare Part A and Part B records.

Data Sharing Plan

After the main results of this study have been published, de-identified data from this study may be shared with other VA investigators, other Federal health agencies, or academic institutions for the purpose of additional analyses provided this use has been approved by the appropriate VA oversight committee and there is an agreement in place that defines the limits of this use.

© 2021 Zenati MA et al. *JAMA Network Open.*
