# Supplementary material for: Intermediate-Term Outcomes of Endoscopic or Open Vein Harvesting for Coronary Artery Bypass Grafting: The REGROUP Randomized Clinical Trial
Source: JAMA Netw Open. 2021 Mar 15;4(3):e211439. doi: 10.1001/jamanetworkopen.2021.1439 (PMC7961312; doi:10.1001/jamanetworkopen.2021.1439)
Supplement: Supplement 3. — Data Sharing Statement [file jamanetwopen-e211439-s003.pdf]

Data Sharing Statement

Zenati. Intermediate-Term Outcomes of Endoscopic or Open Vein Harvesting for Coronary Artery Bypass Grafting. *JAMA Netw Open*. Published March 15, 2021. doi:10.1001/jamanetworkopen.2021.1439

Data
Data available: Yes
Data types: Other (please specify)
Additional Information: De-identified data from this study may be shared with other VA investigators, other Federal health agencies, or academic institutions for the purpose of additional analyses provided this use has been approved by the appropriate VA oversight committee and there is an agreement in place that defines the limits of this use
How to access data: De-identified data from this study may be shared with other VA investigators, other Federal health agencies, or academic institutions for the purpose of additional analyses provided this use has been approved by the appropriate VA oversight committee and there is an agreement in place that defines the limits of this use
When available: With publication

Supporting Documents
Document types: None

Additional Information
Who can access the data: Other VA investigators, other Federal health agencies, or academic institutions.
Types of analyses: Additional analyses.
Mechanisms of data availability: After approval by the appropriate VA oversight committee and there is an agreement in place that defines the limits of this use.
